# Editorial: Sport and Psychosocial Health/Well-Being After the COVID-19 Lockdown

**DOI:** 10.3389/fspor.2022.902973

**Published:** 2022-04-26

**Authors:** Amy Chan Hyung Kim, James Du, Rochelle Eime

**Affiliations:** ^1^Department of Sport Management, Florida State University, Tallahassee, FL, United States; ^2^Institute for Health and Sport, Victoria University, Melbourne, VIC, Australia; ^3^Institute of Health and Well-Being, Federation University, Ballarat, VIC, Australia

**Keywords:** COVID-19, mental health, physical activity, active lifestyle, psychological wellbeing, mental wellbeing, social wellbeing

Since late 2019, the COVID-19 pandemic has significantly impacted one's active lifestyle (Kim et al., 2022). On the one hand, social distancing/isolation restrictions prevented the public from being physically active in that sports events were suspended, postponed, or paused. Sports facilities such as senior centers, recreation centers, and fitness centers were closed, and school-based extracurricular physical activity and sport programs were canceled (Du et al., 2021). On the other hand, new opportunities for active lifestyles emerged. Individual home-based training using technologies such as cycling equipment with virtual roads and virtual classes has boomed. In addition, outdoor-based activities such as hiking, walking, or backpacking have become more popular.

Whilst sport play and competitions were canceled individuals turned to other non-sporting activities. The articles in this Research Topic included the psychosocial health and wellbeing of participation in a range of different activities including sport, non-sporting activities and nature-based outdoor activities. Further, the research studies included a range of different population groups including general population, elite athletes, Paralympic athletes, patients with health conditions and sports volunteers. Below provides a summary of the Research Topic articles.

This Research Topic is about investigating the impact of the COVID-19 pandemic on the association between these (un)changed active lifestyles (e.g., formats, involvement levels, patterns) and psychosocial outcomes of the COVID-19 pandemic from a public health perspective. Authors representing various academic disciplines, such as sport management, sport science, health and behavioral science, and medicine have contributed to this Research Topic by introducing empirical findings, and methodological innovations. In this Editorial, we offer an overview of the diverse contents of the present Research Topic.

Several empirical studies examined various types of physical activity, and one's psychosocial health and wellbeing during and after the COVID-19 restrictions across the world. For example, Wendtlandt and Wicker investigated how nature-based, natural resource-using, and nature-neutral sport activities and different types of environmentally sustainable behaviors, including recycling, ecological consumption, energy-saving, and mobility were associated with subjective wellbeing before and during the COVID-19 lockdown in Germany. The findings showed that nature-based and nature-neutral sport activities significantly decreased during the first COVID-19 lockdown in Germany, while environmentally sustainable behaviors increased. Notably, a decrease in nature-based and nature-neutral sport activities significantly predicted a decline in individuals' subjective wellbeing.

Cindrich et al. explored the impact of COVID-19 restrictions on spending time outside April 2020 and how it was associated with stress and positive mental health in the United States. The findings reported that participants with increased or maintained outside time reported a lower level of stress and a higher level of positive mental health compared to those who decreased outside time, implying being active outside would benefit one's psychological well-being.

Nagarathna et al. investigated if yoga is beneficial for physical and mental health and assessed the lifestyle of yoga practitioners that may be critical in coping with stress was associated with COVID-19 lockdown in India. The findings showed that yoga practitioners reported good physical ability and endurance, lower levels of anxiety, stress, and fear than the non-Yoga group. Additionally, the yoga group showed a better ability to cope with the stress associated with lockdown and COVID-19 than the non-Yoga group.

Some articles examined the influence of COVID-19-related restrictions and lockdowns on individuals psychosocial health and wellbeing status among specific populations, including patients with major depression, infertile women, children with neurodevelopmental disorders, Paralympic athletes, and intercollegiate student-athletes. Cody et al. explored whether data gathered before and during/after the lockdown in Switzerland among in-patients with major depression would be different in terms of psychosocial health, physical activity, and related attitudes. The findings indicated no differences between before and during/after the COVID-19 in terms of psychosocial health, including stress, sleep quality, and both physical and mental elements of health-related quality of life, self-reported physical activity. Cao et al. studied infertile women to investigate their anxiety levels in the quarantined and non-quarantined areas in China during the COVID-19 pandemic. The results showed that quarantined participants tended to have more negative emotions and worse family relationships compared to non-quarantined participants. Ueda et al. studied children with neurodevelopmental disorders who had altered sleep patterns and its effect on quality of life in Japan. The results showed that ~50% of participants experienced changes in sleep patterns. The changes were associated with decreased quality of life, which resulted in a higher level of depression and lowered current mood status.

Hu et al. investigated the athlete identity of Paralympic athletes during the COVID-19 in the United States. Even during the training and competition cessation resulting from the COVID-19 pandemic, athletes with a strong athlete identity tended not to be affected by the pandemic. Floyd et al. explored changes of sentiment in student-athletes at the Division I institutions in the United States during the COVID-19. The findings of machine-learning-based natural language processing techniques of the user-generated Twitter posts suggested that positive sentiment outweighed negative sentiment overall, whereas there was a noticeable spike in negative sentiment in May and June 2020.

Power and Nedvetskaya investigated the theory-practice divide in the volunteer-management relationship in the United Kingdom and how it may impact volunteer experiences and volunteer program outcomes during the COVID-19 pandemic. While the results showed that most research participants expressed an intent to return to sport event volunteering post-pandemic, the authors highlighted some sociodemographic factors such as age or the level of safety protocol might impact future participation in volunteering.

Lastly, You et al. reviewed exercise intervention studies in treating depression among teenagers between 2000 and 2020. The authors identified four future directions in the area of this study: research on the effect of specific exercise intervention, research on the essence of exercise and sports, research on the combination mode of exercise and other elements, and research on the micro and molecular level.

The articles published in this Research Topic are positioned across the various academic fields embracing a broad range of themes related to active lifestyle and mental health during the COVID-19 pandemic.

## Author Contributions

All authors listed have made a substantial, direct, and intellectual contribution to the work and approved it for publication.

## Conflict of Interest

The authors declare that the research was conducted in the absence of any commercial or financial relationships that could be construed as a potential conflict of interest.

## Publisher's Note

All claims expressed in this article are solely those of the authors and do not necessarily represent those of their affiliated organizations, or those of the publisher, the editors and the reviewers. Any product that may be evaluated in this article, or claim that may be made by its manufacturer, is not guaranteed or endorsed by the publisher.
